# Validation of a Novel Patient-Reported Measure of The Burden of Digital Care in Diabetes

**DOI:** 10.21203/rs.3.rs-7265768/v1

**Published:** 2025-08-20

**Authors:** Misk Al Zahidy, Jennifer L. Ridgeway, Megan E. Branda, Kerly Guevara Maldonado, Sue Simha, Alexandra Herzog, Jada Hougen, Mariana Borras-Osorio, Viet-Thi Tran, Victor M. Montori

**Affiliations:** Mayo Clinic; Mayo Clinic; Mayo Clinic; Mayo Clinic; Mayo Clinic; Mayo Clinic; Mayo Clinic; Mayo Clinic; Mayo Clinic; Mayo Clinic

**Keywords:** Diabetes Mellitus, Patient-Reported Outcome Measures, Treatment Burden, Digital Medicine Tools, Chronic Disease Management, Self-Management, Validation Study, Burden of Digital Care

## Abstract

**Background:**

Patients living with diabetes and chronic conditions may face significant burden managing their health. Many of these patients use digital medicine tools such as continuous glucose monitoring systems. Although measures exist to assess treatment burden from tasks such as managing medications and attending healthcare visits, there is no patient-reported measure that captures the burden of digital care. Therefore, the purpose of this study is to validate the Treatment Burden Questionnaire Plus Digital (TBQ + D), a patient-reported measure of treatment burden that includes using digital tools for adults with diabetes.

**Methods:**

Adult patients with type 1 or type 2 diabetes mellitus completed the 25-item TBQ + D (scored 0 [none] to 10 [maximum] per item; total score range 0–250). We evaluated ease of administration, internal consistency, and tested hypotheses about the relationship between TBQ + D scores and treatment complexity, digital tool use intensity, social risk factors, and digital comfort to assess TBQ + D’s validity.

**Results:**

Of 324 patients approached, 300 (93%) consented and completed the TBQ + D (mean age 57 [SD 17]; 50% female; 50% with type 2). The mean TBQ + D score was 53.7 (SD 41.6). Internal consistency was excellent (Cronbach’s α = 0.94). As hypothesized, higher TBQ + D scores were reported by patients with type 1 vs. type 2 diabetes mellitus (61.7 vs. 45.7; p = .0008), maximal/moderate vs. minimal to no digital tool use (56.5/60.7 vs. 41.3; p = .001), those on intensive insulin therapy vs. other treatments (61.4 vs. 38.0; p < .0001), and those with greater social vulnerability (p < .0106). TBQ + D scores were not significantly higher in patients with HbA1c ≥ 8% (p = .055) or less comfortable with digital technology (p = .08).

**Conclusions:**

TBQ + D is a novel and valid measure of treatment burden in patients living with diabetes, inclusive of digital burden, that can play a role in fostering minimally disruptive care for patients with diabetes.

## BACKGROUND

Patients living with chronic conditions, such as diabetes mellitus, invest substantial time, energy, financial resources, and attention to access and use healthcare, and perform ongoing self-management tasks.(1–6) These tasks include monitoring blood glucose, implementing insulin dosing, managing food choices and exercise, and coordinating appointments with the healthcare system.(7–9) The cumulative demand of this work and its negative impact on a patient’s quality of life is referred to as treatment burden.(7, 10–13) Higher treatment burden is associated with decreased adherence to care plans, increased hospitalization rates, and overall poorer health outcomes.(7, 14)

Recognizing these challenges, there has been a shift in chronic disease management for patients living with diabetes over the past decade enabled by the proliferation of useful, usable, and desirable digital medicine devices and applications supporting self-monitoring and self-management.(15–19) These technologies include patient portals, virtual visits, continuous glucose monitoring systems, automated insulin delivery systems, and mobile self-management applications.(16, 18–22) These tools have the potential to alleviate the workload by streamlining or automating many of the routine tasks required for disease management.

Despite these benefits, digital medicine tools can also inadvertently exacerbate treatment burden.(23, 24) Usability issues, information overload, frequent alerts, technical malfunctions, and privacy concerns can increase the overall workload for patients, particularly those less familiar with or with limited access to digital technology, as highlighted in our previous work.(25–28)

Multimorbidity is highly prevalent among patients with type 2 diabetes,(29) and it has fueled the development, validation, and adoption of disease-generic instruments to self-report treatment burden. Instruments such as the Treatment Burden Questionnaire (TBQ) and Patient Experience with Treatment and Self-management,(5, 11, 14, 23, 30, 31) are among the most widely used, but they were not designed to capture burden specifically related to the adoption and routine use of digital medicine tools. This gap hinders our ability to fully understand and address the challenges patients face in the digital age of healthcare.(25, 32)

To address this gap, our team has previously conducted patient interviews to adapt the TBQ to include items specific to digital devices and applications, resulting in the Treatment Burden Questionnaire Plus Digital (TBQ + D), the first instrument specifically designed to assess all aspects of treatment burden including the burden of digital medicine tools.(25, 33)

The aim of this study is to validate the TBQ + D by assessing its feasibility of administration, internal consistency, and construct validity. Construct validity was evaluated by testing hypotheses of association between TBQ + D scores and patient factors expected to contribute to greater treatment burden in adult patients living with diabetes.

## METHODS

This study represents the final phase in the development of the TBQ + D. The initial phases, concept elicitation interviews to explore the burden of digital tool use and iterative tool development through item generation and cognitive testing, have been reported previously.(25, 33)

### Participants and recruitment

Adult patients aged 18 and older with a diagnosis of type 1 or type 2 diabetes and attending the diabetes clinic in the Division of Endocrinology at Mayo Clinic (Rochester, Minnesota) were eligible to participate if they used at least one digital medicine tool for diabetes management (e.g. glucometer, insulin smartpens, pumps) (**Additional file 1**). Patients who could not give consent (unable to communicate in English or with significant cognitive or sensory impairments) or whose caregivers were the primary users of digital tools were excluded. Study staff identified eligible patients from the daily appointment list and approached them in-person. We targeted a sample size of 300 participants, a commonly recommended minimum for scale validation and subgroup analyses, and, given our clinic’s volume (> 500 eligible patients per week), this was feasible within our time and budget constraints.

Recruitment aimed to maximize diversity in age, sex, diabetes mellitus type (type 1 or type 2), glycemic management (measured by HbA1c), and digital tool use intensity, which was measured using the Digital Medicine Tools Intensity Scale—a 7-point scale ranging from “none” to “maximal” (**Additional file 2**). To ensure a diverse sample and minimize selection bias, participant characteristics were reviewed by the study statistician (M.E.B.) after every 50 enrolled patients. Based on these reviews, the statistician provided input on sample distributions and recommended adjustments to recruitment priorities (e.g., targeted outreach for underrepresented groups such as patients with type 1 diabetes or those with low digital tool use).

### Data sources

To assess construct validity, participants were invited to complete the 25-item TBQ + D (Table 1), the Unified Theory of Acceptance and Use of Technology (UTAUT) scale (to capture digital comfort)(34), and the MacArthur Scale of Subjective Social Status(35) (to capture social vulnerability) in a private room prior to their clinic appointment. Sociodemographic and clinical data—including age, gender, diabetes type, HbA1c levels, and hypoglycemia history—were extracted from the electronic health record.

#### Data Analysis

The TBQ + D was assessed in terms of feasibility of administration, internal consistency, and construct validity. Feasibility of administration was quantified as the proportion of eligible patients who completed the questionnaire and the proportion of missing items in their responses.

To assess the variability of responses for each item, we estimated means, standard deviations, and frequency distributions for each TBQ + D item. Items with limited variability (≥ 95% of responses identical) could be considered for exclusion due to low discriminatory power. Total scores were calculated by summing responses across the 25 items, each scored from 0 (no burden) to 10 (maximum burden), for a possible range of 0 to 250. Responses marked as “Does not apply” were retained and scored as zero to reflect no burden in that domain when the total score was calculated.

Internal consistency was assessed using Cronbach’s α with values between 0.70 and 0.95 considered acceptable.(36)

We then tested six key construct validity hypotheses based on the Cumulative Complexity Model,(37, 38) an evidence-based framework that describes treatment burden as resulting from an imbalance between the workload a patient must shoulder to enact self-care tasks and the capacity they have to do so. In this model, burden increases when workload exceeds capacity.

Based on the framework, we hypothesized that TBQ + D scores would be higher among patients facing greater workload (e.g., high digital treatment intensity and intensive insulin therapy) and/or lower capacity (e.g., adverse social risk factors, hyperglycemia, and reduced digital comfort). To test these hypotheses, we compared the TBQ + D total scores across patient groups based on HbA1c levels (< 8% vs. ≥8%), use of intensive insulin therapy (e.g., basal-bolus regimen, continuous insulin infusion) vs. other treatments, social vulnerability (MacArthur Scale of Subjective Social Status), and digital comfort (UTAUT subscales: performance expectancy, effort expectancy, social influence, and facilitating conditions). We used t-tests or Kruskal-Wallis tests, as appropriate, for group comparisons based on distribution and number of categories, and Spearman’s rank correlation coefficients to assess associations with continuous or ordinal variables, with r > 0.50 considered strong and 0.35–0.50 considered moderate.

All statistical analyses were performed using SAS (version 9.4; SAS Inst., Cary, NC, USA). Participant responses, demographic, and clinical data were managed using Research Electronic Data Capture (REDCap), a secure, web-based software platform hosted at Mayo Clinic (UL1TR002377) designed to support data capture for research studies.(39) Missing data were not imputed.

### Ethical consideration

The Mayo Clinic Institutional Review Board (IRB Number 23–007631) approved all procedures. Informed consent was obtained from each participant using an oral consent script alongside a standalone Health Insurance Portability and Accountability Act (HIPAA) authorization for records review.

## RESULTS

### Participant characteristics

Field testing and validation took place between May and October 2024. Of 324 patients approached, 300 (92.6%) consented and completed the TBQ + D (mean age 57 years, SD = 17; 50% female; 50% with type 2 diabetes; Table 2), and 24 declined due to time constraints. A range of digital tool use intensities was represented, with 103 (34%) participants classified as maximal-intensity users.

### Feasibility of administration

The average time to complete the TBQ + D was 5 minutes (SD = 2.8). Item-level missingness was minimal, with no item missing from more than six responses (**Additional file 3**). The overall item-level completion rate was 99%, indicating high feasibility of administration.

All TBQ + D items were applicable to most participants, with the proportion of “does not apply” responses ranging from 0–22%, and an average of 2.7% across items. The highest non-applicability was observed for the item regarding the taste, shape, or size of pills (22%). None of the items demonstrated low variability (≥ 95% identical responses). Response distributions are summarized in **Additional file 4** and visualized in **additional file 5**.

### Internal consistency of TBQ + D

The internal consistency (Cronbach’s α) for the overall TBQ + D scale was 0.94.

### Construct validity of TBQ + D

As hypothesized, we found that participants with type 1 diabetes reported higher (p = .0008) TBQ + D scores (mean 61.7, SD 42.3) than patients with type 2 diabetes (45.7, SD 39.4). Participants using intensive insulin therapy reported significantly higher (p < .0001) TBQ + D scores (61.4, SD 41.9) than patients receiving other diabetes treatments (37.8, SD 36.3). Participants with maximal (56.5, SD 37.3) and moderate digital tool use intensity (60.7, SD 45.7) reported significantly higher (p = .003) TBQ + D scores compared to those with minimal or no digital tool use (40.3, SD 37.9).

We also hypothesized that patients with diminished capacity to shoulder the work of being a patient would experience greater treatment burden. Compared to patients reporting higher social status (50.4, SD 39.2), participants reporting lower social status had significantly higher (p = .01) TBQ + D scores (65.2, SD 47.8).

Patients with hyperglycemia (HbA1c ≥ 8%) reported higher TBQ + D scores (61.1, SD 40.7) than those with HbA1c < 8% (50.8, SD 42.5); this difference was not significant (p = .055). We also hypothesized that patients less comfortable using digital tools (UTAUT Scores) would experience higher TBQ + D scores. However, none of the UTAUT subscales showed significant correlations with TBQ + D scores (r) across subscales. Correlations ranged from − 0.113 to 0.08, with r = − 0.071 for the overall composite score (p = 0.22).

## DISCUSSION

This study supports the TBQ + D as a feasible, internally consistent, and valid novel patient-reported instrument designed to assess treatment burden—including the burden introduced by digital medicine tools—in adults living with diabetes. As hypothesized, higher burden scores were observed in patients facing greater treatment complexity (e.g., type 1 diabetes, intensive insulin therapy), greater digital tool use, and greater social vulnerability. Contrary to our expectations, we did not find greater burden in patients with hyperglycemia or less comfortable with digital tools.

These findings align with prior research highlighting the burden associated with digital medicine tools, emphasizing the importance of designing digital interventions that support patients without adding to their workload.(16, 40–43) A recent scoping review on digital health technologies in multimorbidity management has identified key mechanisms by which digital tools affect patient care, including care coordination, self-management, and remote monitoring.(44) Our previous work further expands upon this by identifying areas in which digital medicine tools can either alleviate or exacerbate treatment burden.(25, 33, 45)

Our study advances the field through the development of what may be the first self-reported, disease-generic instrument to measure digital treatment burden. In this manner, TBQ + D represents a critical step toward ensuring that digital health interventions contribute to patient-centered care without unduly increasing the burden of care.

Our findings have several implications for clinical practice and the integration of digital medicine tools into routine care. First, the TBQ + D provides a valid and reliable instrument to measure digital treatment burden among patients with diabetes, allowing researchers to explore how digital burden can be systematically assessed and addressed within clinical workflows. Second, although we considered exploratory and confirmatory factor analyses during development, we deliberately chose not to report those models here: the fit indices were poor in our sample and, more importantly, factor-analytic solutions did not align with our patient-centered focus on item relevance; future work with larger, independent cohorts and patient-rated importance data will revisit dimensional validity. TBQ + D can also enable the assessment of the effects that digital tools can have on patients’ daily lives. As healthcare increasingly relies on digital medicine for chronic disease management, it is important to ensure that these tools support care and enable flourishing while exerting minimal adverse effects on quality of life.

Patients with higher treatment complexity may experience a greater digital workload, underscoring the need for an individualized approach to their use.(46) Clinicians should assess not only digital literacy but also the cumulative burden imposed by digital interventions, ensuring that digital tools simplify care rather than introduce unnecessary complexity.(23) Assessing factors, such as mental health comorbidities and relational difficulties (12, 14), that contribute to persistently elevated burden levels or to levels that increase over time can helpfully orient supportive interventions to minimize the burden of treatment towards patients at the highest risk of becoming overwhelmed by self-management and healthcare demands.

Shared decision-making may be central to the integration of digital medicine tools into care plans, ensuring that these tools align with the needs, capabilities, and daily routines of each patient.(47) To this end, digital tools should be designed and implemented in ways that seamlessly fit into patients’ lives rather than adding unnecessary complexity or burden.(24) Routine use of the TBQ + D could spur the design of digital interventions that are easy to adopt and use and of implementation protocols that include effective, targeted, and tailored support.

This initial evaluation, testing a parsimonious, theory-driven set of hypotheses, advances the evidence of construct validity of the TBQ + D. However, out findings may not apply to more diverse and disadvantaged populations in primary care settings. While conducted with patients with diabetes, our study population demonstrated multimorbidity common among older patients. As additional evidence accrues, the TBQ + D may ultimately assist researchers and clinicians in assessing the real-world impact of digital tools among patients with multimorbidity and in evaluating interventions developed to mitigate treatment burden.

Whether TBQ + D validly measures the overall burden of treatment, including digital burden, in patients with substantial multimorbidity but mild diabetes also awaits further exploration. Validity hypotheses relating changes in personal circumstances, health status, and treatment plans (including the adoption of additional digital tools) to changes in TBQ + D scores could not be tested using this cross-sectional design; its responsiveness to change so that it can be used as an outcome measure in randomized trials of minimally disruptive medicine interventions remains to be established.

## CONCLUSIONS

In conclusion, we present evidence of the internal consistency and construct validity of the TBQ + D as a patient-reported measure of the burden of treatment that considers the negative effect of both conventional and digital workloads on the quality of life of patients with diabetes. In doing so, it contributes to the implementation of evidence-based, minimally disruptive care regimens that fit each person living with diabetes.

## Supplementary Material

Supplementary Files

This is a list of supplementary files associated with this preprint. Click to download.

- Additionalfile1.pdf
- Additionalfile2.pdf
- Additionalfile3.pdf
- Additionalfile4.pdf
- Additionalfile5.pdf

## Figures and Tables

**Table 1:** TBQ + D items

Item no.	Item	Mean (SD)
1	How much of a problem is the taste, shape, or size of your pills?	1.1 (2.36)
2	How much of a problem are the annoyances caused by your injections (For example: bleeding, bruising, or scarring)?	2.2 (2.77)
3	How much of a problem is the number of times that you should take your medication daily?	1.9 (2.68)
4	How much of a problem are the efforts you make to help you remember to take your medications (For example: managing your treatment when away from home, preparing pillboxes, or setting reminders on your phone)?	2.6 (2.84)
5	How much of a problem is it to take the necessary precautions when taking your medication (For example: taking your medication at specific times of the day or meal or being unable to do certain things after taking medications such as driving or lying down)?	2.3 (2.82)
6	How much of a problem is it to arrange medical appointments (finding and scheduling doctor visits, telehealth visits, lab tests, and other exams) and to reorganize your schedule around these appointments (For example: coordinating fasting blood draws)?	2.3 (2.73)
7	How much of a problem is it to attend doctor’s visits and other office visits: given their frequency and time spent participating in these visits?	1.9 (2.29)
8	How much of a problem is it to complete lab tests and other exams: given their frequency, time spent and associated nuisances or inconveniences (For example: getting your blood drawn or checking your lab results on the patient portal)?	1.6 (2.18)
9	How much of a problem are the difficulties you have in your relationships with healthcare professionals (For example: feeling not listened to enough, not taken seriously, or feeling uneasy or disconnected during telehealth visits)?	1 (25)
10	How much of a problem is the time and effort required for self-monitoring: given their frequency, time spent and associated nuisances or inconveniences (For example: taking your blood pressure or keeping diary for your diet)?	2.5 (2.84)
11	How much of a problem is the administrative burden related to healthcare (For example: managing account details and passwords, and filling out paperwork or online forms for prior authorizations, reimbursements, and device orders or replacements)?	2.7 (2.98)
12	How much of a problem is the financial burden associated with your healthcare (For example: paying for treatments or providers not covered by insurance or out-of-pocket expenses like the cost of devices or device replacements)?	3.1 (3.23)
13	How much of a problem is it to make changes in eating and other habits (For example: avoiding certain foods or alcohol, having to quit smoking…)?	3.7 (2.96)
14	How much of a problem is it to follow doctors’ recommendations to practice physical activity (For example: walking, jogging, swimming…)?	2.5 (2.74)
15	How much of a problem is how your healthcare affects your relationships with others (For example: Having to rely on family and friends for assistance or needing to educate them about your condition)?	1.8 (2.67)
16	How much of a problem is it to manage your health discreetly (For example: checking blood sugar levels or administering injections in public, the visibility of your devices, or concerns about being judged)?	1.9 (2.65)
17	How much of a problem is the privacy and security of your health information (For example: the confidentiality of your health information, managing who can have access to it, sharing your data with insurers in ways that could affect your coverage or claims, or feeling less free to live your life the way you want)?	1.2 (2.25)
18	‘The need for healthcare on a regular basis reminds me of my health problems’.	3.5 (3.41)
Digital Module		
1	How much of a problem is the time and effort required to take care of and use your digital devices in your day-to-day life? (For example: logging on for a telehealth visit, remembering to change sensors or charge devices, syn	2.6 (2.70)
2	How much of a problem is the annoyance and discomfort of your digital devices? (For example: device alarms or notifications interrupting daily activities or difficulty in wearing device with formal attire or beachwear)	3.1 (35)
3	How much of a problem is the time and effort required to solve problems with your digital devices? (For example: troubleshooting a new or old device, or finding educational resources from the healthcare team or device manufacturer)	2.6 (2.82)
4	How much of a problem is it to take the necessary precautions when using your digital devices? (For example: protecting your digital devices from drops, certain temperatures, or radiation (security checkpoint, MRIs, etc.) or carrying backups like a glucometer for continuous glucose monitors, or insulin pens for insulin pumps, etc.)	2.6 (2.79)
5	How much of a problem is it to give control of your health to digital devices? (For example: feeling uneasy or worried about the automatic functions of your device)	1.5 (2.42)
6	‘The need for digital devices (including their visibility) on a regular basis reminds me of my health problems’.	3.5 (3.33)

**Table 2:** Patient characteristics by digital intensity

	Digital tool use intensity				
	Minimal (1) (N = 50)	Low (2, 3) (N = 36)	Moderate (4, 5) (N = 111)	Maximal (6, 7) (N = 103)	Total (N = 300)
Diabetes diagnosis, n (%)					
Type 1 diabetes	2	5	50	93	150 (50%)
Type 2 diabetes	48	31	61	10	150 (50%)
Age at enrollment					
Mean (SD)	64 (14.5)	60 (16.7)	57 (15.9)	52 (18)	57 (16.8)
Female, n (%)	22	10	65	54	151 (50%)
Race, n (%)					
White	41	31	101	95	268 (89.6%)
Black or African American	1	0	4	3	8 (2.7%)
Asian	3	0	1	2	6 (2%)
American Indian or Alaskan Native	0	1	1	1	3 (1%)
Two or more races	1	1	1	2	5 (1.7%)
Other	4	2	3	0	9 (3%)
Missing	0	1	0	0	1
Hispanic or Latino ethnicity, n (%)	4	1	4	1	10 (3.4%)
BMI					
Mean (SD)	29.8 (7.13)	32.8 (10.53)	32.2 (10.97)	28.7 (6.28)	30.7 (9.05)
HbA1c levels					
Mean (SD)	8 (2.08)	7.4 (1.54)	7.8 (1.63)	7.1 (0.96)	7.6 (1.54)
< 7	13 (29.5%)	19 (54.3%)	36 (33%)	44 (44.9%)	112 (39.2%)
7 to < 8	12 (27.3%)	4 (11.4%)	29 (26.6%)	40 (40.8%)	85 (29.7%)
8 to < 9	11 (25%)	5 (14.3%)	21 (19.3%)	9 (9.2%)	46 (16.1%)
9 to < 10	3 (6.8%)	5 (14.3%)	13 (11.9%)	4 (4.1%)	25 (8.7%)
10+	5 (11.4%)	2 (5.7%)	10 (9.2%)	1 (1%)	18 (6.3%)
Missing	6	1	2	5	14
Health insurance, n (%)					
Medicare	23	11	29	31	94 (31.3%)
Medicare advantage	4	4	10	4	22 (7.3%)
Medicaid	1	4	6	3	14 (4.7%)
Private	21	16	65	65	167 (55.7%)
No insurance/self-pay	1	1	1	0	3 (1%)

**Table 3:** Testing the construct validity of the TBQ + D

Variable	Group	Median (IQR)	p value
WORKLOAD HYPOTHESES			
Diabetes Type			.0008
	Type 1	54.0 (26.0, 92.0)	
	Type 2	36.0 (14.0, 63.0)	
Intensive Insulin			< .0001
	No	25.5 (12.0, 59.0)	
	Yes	53.0 (28.0, 89.0)	
Digital Tool Use Intensity			.003*
	Minimal (1)	28.5 (12.0, 55.0)	
	Low (2–3)	30.0 (11.5, 63.5)	
	Moderate (4–5)	58.0 (19.0, 91.0)	
	Maximal (6–7)	50.0 (26.0, 85.0)	
CAPACITY HYPOTHESES			
HbA1c			.055
	< 8%	39.0 (16.0, 75.0)	
	≥ 8%	59.0 (28.0, 86.0)	
Social Status			0.01
	High	43.0 (18.0, 75.0)	
	Low	51.0 (32.0, 97.0)	
Digital Comfort			.08
	≤ 3	28.5 (12.0, 63.5)	
	> 3	46.0 (22.0, 83.0)	

* All hypotheses were tested using t-test except for digital tool intensity which used Kruskal-Wallis test.

## Data Availability

The datasets generated and/or analyzed during the current study are not publicly available due to protections around participant privacy but are available from the corresponding author on reasonable request and subject to appropriate data use agreements.

## Abbreviations

HIPAA: Health Insurance Portability and Accountability Act
REDCap: Research Electronic Data Capture
TBQ+D: Treatment Burden Questionnaire Plus Digital
UTAUT: Unified Theory of Acceptance and Use of Technology
